# Supplementary material for: Extrapyramidal side effects of antipsychotics are linked to their association kinetics at dopamine D2 receptors
Source: Nat Commun. 2017 Oct 2;8:763. doi: 10.1038/s41467-017-00716-z (PMC5624946; doi:10.1038/s41467-017-00716-z)
Supplement: Supplementary file 1 — Supplementary Information [file 41467_2017_716_MOESM1_ESM.pdf]

Description of Supplementary Files

File name: Supplementary Information
Description: Supplementary figures and supplementary references.

File name: Peer review file

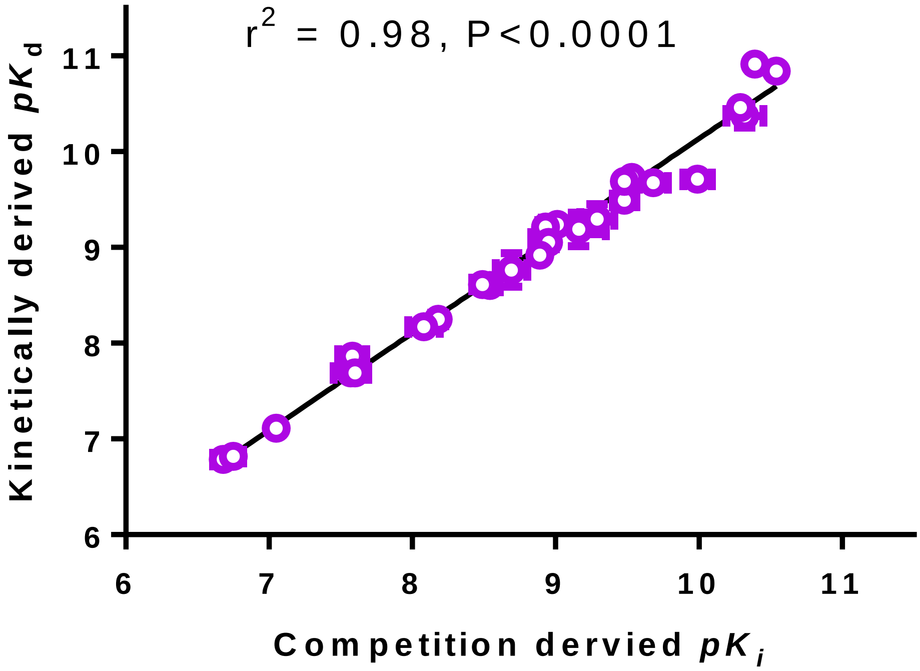

Supplementary Figure 1. Correlating equilibrium and kinetically derived parameters of D_2 atypical and typical AP ligands. Correlation between pK_i and kinetically derived pK_d for the 27 test ligands. pK_i values were taken from PPHT-red competition binding experiments at equilibrium as exemplified in Figure 2A. The values composing the kinetically derived pK_d (k_{off}/ k_{on}) were taken from competition kinetic association experiments as exemplified in Figure 2B-D. All data used in these plots are detailed in Table 1. Data are presented as mean ± S.E.M. from four separate experiments.

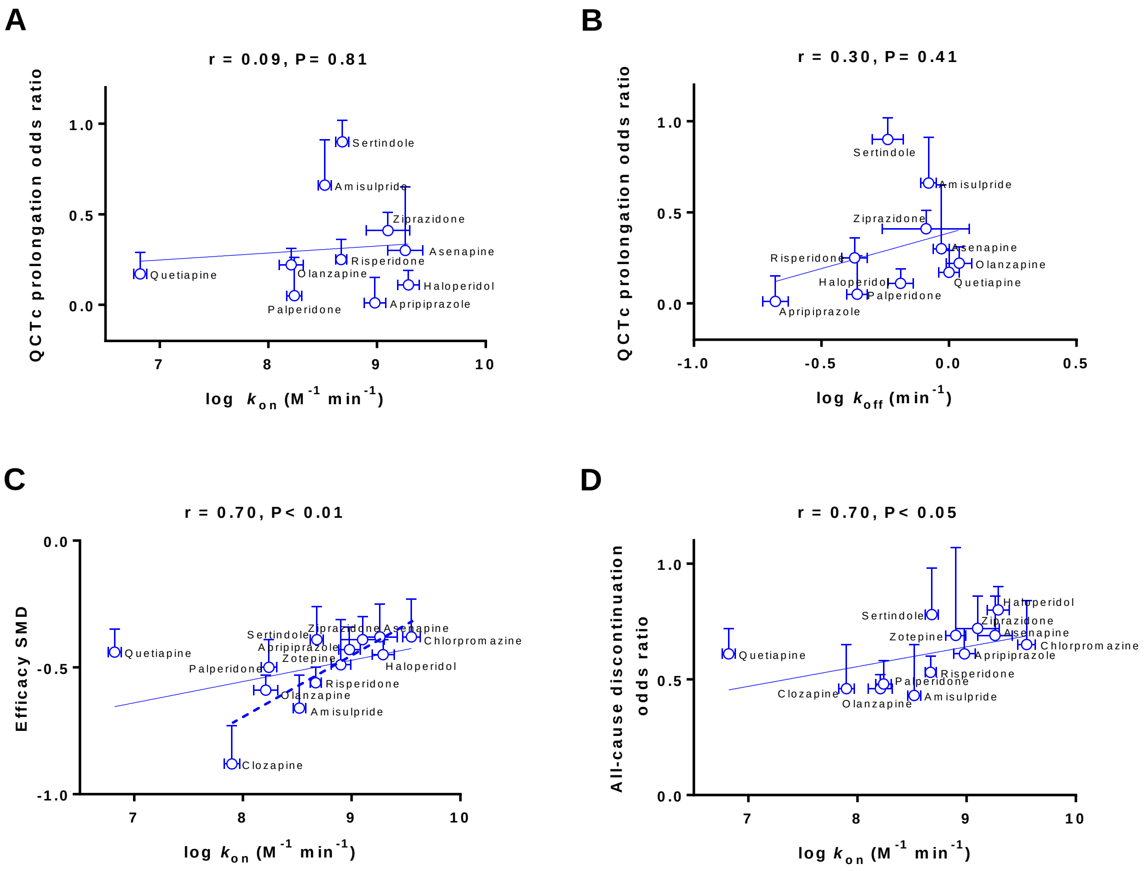

Supplementary Figure 2. Correlating clinical data on APD efficacy, all cause discontinuation and 'off target' effects with kinetically derived parameters. Correlation plot showing the relationship between (A) log k_{on} and QCTc prolongation odds ratio and (B) log k_{off} and QCTc prolongation odds ratio. Correlation plot showing the relationship between log k_{on} and (C) efficacy measure based on mean overall change in symptoms and log k_{on} and (D) all cause discontinuation odds ratio. All kinetic data used in these plots are detailed in Table 1 and clinical data are taken from Leucht *et al.*[1] Kinetic data are presented as mean ± S.E.M. from four experiments and clinical data as odds ratios with associated credible intervals.

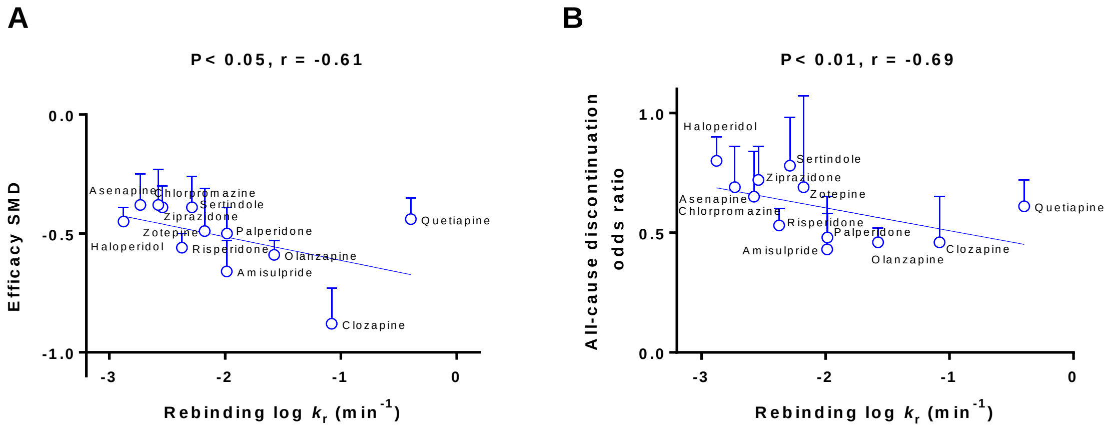

Supplementary Figure 3. Relationship between APD receptor D_2R rebinding and measures of clinical efficacy and all-cause discontinuation. Correlation plot showing the relationship between (A) log k_r and efficacy measures based on mean overall change in symptoms and (B) log k_r and all-cause discontinuation odds ratio. All kinetic parameters used to produce these plots are detailed in Table 1 and in the methods section associated with equation (4). Clinical data are taken from Leucht *et al.*[1]. For simulation purposes the rebinding reversal rate k_r was based on the model of an immunological synapse. Kinetic data are presented as mean ± S.E.M. from four experiments and clinical data as odds ratio with associated credible intervals.

Supplementary references

1. Leucht, S. *et al.* Comparative efficacy and tolerability of 15 antipsychotic drugs in schizophrenia: a multiple-treatments meta-analysis. *Lancet* **382,** 951-962 (2013).
